# Factors associated with different patterns of weight change after bariatric surgery: A longitudinal study

**DOI:** 10.1002/osp4.675

**Published:** 2023-04-28

**Authors:** Diana Cristina Henao Carrillo, Ana María Gómez, Oscar M. Muñoz, Claudia Rubio, Natalia Rodríguez, Valentina Ursida, Ana Milena Forero, Fabio Pinzón, Rami Mikler

**Affiliations:** ^1^ Pontificia Universidad Javeriana Bogotá Colombia; ^2^ Endocrinology Unit Hospital Universitario San Ignacio Bogotá Colombia; ^3^ Internal Medicine Department Hospital Universitario San Ignacio Bogotá Colombia; ^4^ Surgery Department Hospital Universitario San Ignacio Bogotá Colombia; ^5^ Nutrition Department Hospital Universitario San Ignacio Bogotá Colombia

**Keywords:** bariatric surgery, generalized estimating equation, obesity, weight loss

## Abstract

**Background:**

The mean weight loss (WL) after successful bariatric surgery is approximately one third of the initial body weight, which is mainly achieved between the first 2 years of follow‐up. However, 15%–35% of patients do not achieve a significant percentage of total WL (%TWL). Information on factors associated with a higher or lower WL after bariatric surgery is limited. This study aimed to assess the change in %TWL and describe the factors associated with greater or lesser WL over time.

**Methods:**

This prospective longitudinal study included patients treated with laparoscopic Roux‐en‐Y gastric bypass or sleeve gastrectomy. Baseline data were recorded before surgery. Follow‐up was performed at 3 (*n* = 141), 6 (*n* = 208), 9 (*n* = 115), 12 (*n* = 216), 24 (*n* = 166), and 36 months (*n* = 99). Generalized estimating equation analysis was performed to assess the changes in %TWL over time and factors associated with different patterns of WL.

**Results:**

In total, 231 patients were included (women, 82.2%; basal body mass index (BMI) 41.4 ± 5.1 kg/m^2^). The tendencies to increase %TWL (32 ± 6.5) were evident in the first year and stabilized thereafter. Sustained nutritionist follow‐up (2.3%, *p* = 0.004), baseline BMI >40 kg/m^2^ (0.4%, *p* < 0.001), and WL ≥ 10 kg before surgery (0.3%, *p* = 0.001) were associated with a higher %TWL. Patients who performed physical activity >30 min/day after surgery reduced their %TWL by 0.6% (*p* = 0.002).

**Conclusions:**

Modifiable factors such as nutritional monitoring and WL before surgery are associated with a significant increase in %TWL over time. Basal BMI was associated with a significant decrease in %TWL.

## Graphical abstract

Follow‐up during the first year after bariatric surgery is critical to achieving the percentage of total weight loss (%TWL) goals. The factors associated with a greater or lesser WL after bariatric surgery have been limited by inadequate and incomplete long‐term follow‐up. Modifiable factors such as nutritional monitoring and WL before surgery are associated with a significant increase in %TWL over time. Basal BMI was associated with a significant decrease in %TWL.

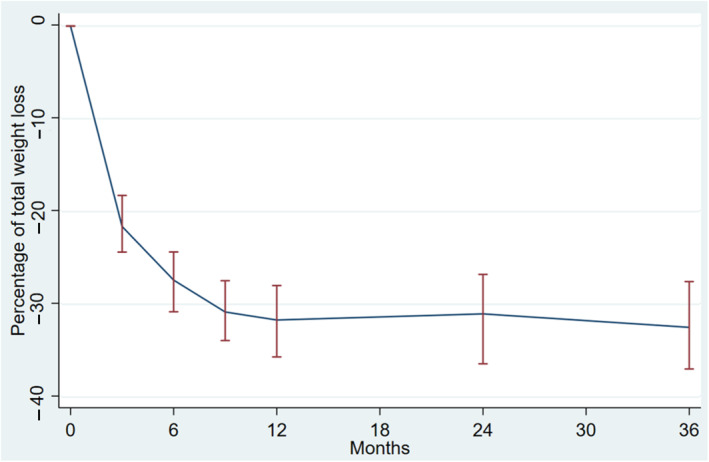

**Abbreviations**
AHI: apnea‐hypopnea index
BMI: body mass index
GEE: generalized estimating equation
HbA1c: glycated hemoglobin A1c
IBW: ideal body weight
RYGB: laparoscopic Roux‐en‐Y gastric bypass
SD: standard deviation
T2D: type 2 diabetes
WL: weight loss
%EWL: percentage of excess weight loss

## 1 INTRODUCTION

Obesity is a chronic, prevalent, and multifactorial disease.^1^ It is associated with an increased risk of type 2 diabetes, dyslipidemia, sleep apnea, coronary heart disease, deterioration in quality of life and increased mortality.^1^, ^2^, ^3^ Bariatric surgery achieves rapid, significant, and sustained weight loss (WL) as well as remission of obesity‐related comorbidities and overall mortality.^4^, ^5^, ^6^ Observational studies have reported that the greatest WL occurs in the first 2 years after bariatric surgery.^7^ However, 15%–35% of patients do not reach their WL goal in the first 2 years after the procedure.^4^

The WL trajectory after bariatric surgery is affected by clinical, anthropometric, and demographic factors such as high baseline body mass index (BMI), gender, age, race, hypertension, hepatic steatosis, sleep apnea, and diabetes.^8^, ^9^, ^10^ Several additional predictors of poor WL have been described: neural factors such as hypothalamus and nucleus accumbens activity during the enhancement of food palatable desire,^11^ psychological factors, such as problematic eating behaviors and psychological distress, and clinical factors, such as glycated hemoglobin A1c (HbA1C).^12^ Meanwhile, adherence to diet and exercise after surgery have been described as positive prognostic factors.^4^ However, the evidence is conflicting and comes mainly from the Caucasian population.

The evidence on the factors associated with a greater or lesser WL after bariatric surgery has been limited by inadequate and incomplete long‐term follow‐up, especially in Latin America. This information would be useful to find predictors related to greater or lesser WL during follow‐up. Therefore, this study aimed to identify the factors associated with different patterns of WL in a cohort of patients who underwent bariatric surgery at an obesity clinic in Latin America.

## 2 MATERIALS AND METHODS

### 2.1 Study design and population

In this prospective longitudinal study, we included adults treated with laparoscopic Roux‐en‐Y gastric bypass (RYGB) or laparoscopic sleeve gastrectomy who were followed up at the Obesity Clinic of Hospital Universitario San Ignacio in Bogotá, Colombia, between January 2011 and December 2019. Patients with a history of surgical complications such as peritonitis, anastomotic fistula formation, thrombotic complications (superior mesenteric vein thrombosis, pulmonary embolism, and deep vein thrombosis), or infection in the immediate postoperative period were excluded. Patients with a glomerular filtration rate ≤30 ml/min/1.73 m^2^, CHILD C liver cirrhosis or liver failure, alcoholism or drug dependence, and pregnancy or active neoplasms were also excluded.

### 2.2 Data collection

Data on baseline demographics, the type of surgery, comorbidities, and clinical characteristics, such as preoperative weight and the BMI, were obtained from the systematically collected medical records. The lipid profile was measured upon admission, and the criteria of the 2019 European Society of Cardiology/European Atherosclerosis Society guidelines were used to diagnose dyslipidemia.^13^ Sleep apnea was confirmed by polysomnography findings. The HbA1c level was measured at baseline using the standardized method recommended by the American Diabetes Association (high‐performance liquid chromatography). Type 2 diabetes was defined according to the American Diabetes Association diagnostic criteria^14^ and non‐alcoholic steatohepatitis documented on liver ultrasonography. A single calibrated electronic column scale (Seca 703) with a load capacity of 300 kg was used to measure weight in all patients at each visit.

During the first year, the follow‐up was performed at 3, 6, 9, and 12 months by a multidisciplinary team that included a bariatric surgeon, nutritionist, and endocrinologist. Subsequently, waist circumference and weight measurements were performed annually until the third year. Prescription for proteins, micronutrients, and a vitamin D supplement was provided to all patients during the first year. Vitamin D and micronutrient supplementation was then continued indefinitely.

A detailed analysis of patients' lifestyle habits was conducted using questionnaire‐based interviews performed 12 months after surgery. This questionnaire was designed to assess the type, intensity, and duration of patients' physical activity and alcohol consumption (the amount of alcohol, type of alcoholic beverage, and frequency of alcohol consumption). An adequate physical activity was defined as that performed ≥5 days a week with moderate intensity, that of vigorous intensity ≥3 days a week, or a combination of moderate and vigorous intensity between 3 and 5 days a week, with a minimum time of 200 min per week. Patients who attended at least two nutrition consultations per year were classified as adherents to the nutritional follow‐up, and the number of visitations with the nutritionist during 36 months was recorded in the medical records. Adherence to the diet was defined as the consumption of ≤1200 calories per day and was evaluated using 24‐h recall questionnaires at each visit. At follow‐up visits, the calculation of grams of alcohol was conducted using the formula designed by the World Health Organization: grams of alcohol (g) = volume (cc) × (graduation × 0.8)/100.^15^ The percentage of total weight loss (%TWL) (initial weight—current weight/initial weight × 100)^16^ was calculated at 3, 6, 9, and 12 months and every year after surgery. Ideal body weight was calculated based on the body structure of the Hispanic population as follows: Ideal body weight = 22 × H^2^, where H was equal to patient height in meters.^17^

### 2.3 Statistical analysis

For continuous variables, the mean and standard deviation or median and interquartile ranges are reported according to the variable distribution. For categorical variables, the frequency and percentage are reported. The advantage of the generalized estimating equation (GEE) is that it considers the fact that the serial observations of the same patient are autocorrelated, and it lets us evaluate how the average of the response variables changes with the covariates. Herein, an exchangeable correlation structure was used. As a sensitivity analysis, GEE models were fitted assuming an unstructured or “independent” correlation structure with no significant change in the results. Multivariable GEE analysis was used to identify the coefficients of each covariate. Variables included in the analysis were age, the type of surgery, presence of diabetes, basal BMI, WL before surgery, adherence to diet and continued follow‐up with a nutritionist, anxiety, eating disorders, alcohol consumption, and physical activity. The time model with a significant contribution (*p*‐value <0.05) and the lowest quasi‐likelihood information criterion represent the best model for the data.^18^ The STATA 16.0 statistical package (StataCorp.) was used to perform the analyses. The Ethics Committee of Hospital Universitario San Ignacio approved the protocol, and all methods were conducted in accordance with relevant guidelines and regulations.

## 3 RESULTS

The demographic and clinical data of patients are shown in Table 1. In total, 231 patients were included in the analysis (women, 82.2%; mean age, 54 ± 12.9 years). Sleep apnea (77.4%), dyslipidemia (51.9%), non‐alcoholic steatohepatitis (47.2%), and hypertension (53.6%) were the most frequent comorbidities. Type 2 diabetes was present in one‐third of the patients with a mean HbA1c level of 6.1 ± 1.2%. Almost two‐thirds of patients were treated with RYGB.

**TABLE 1:** Baseline characteristics and postoperative follow‐up findings.

	*N* = 231	
Age in years, mean (DS)	54	(12.9)
Female sex, *n* (%)	190	(82.2)
Anthropometric measures before surgery		
Baseline weight in kg, mean (SD)	104.9	(15.8)
Body mass index in kg/m^2^, mean (SD)	41.4	(5.1)
Waist perimeter in centimeters, mean (SD)	123.2	(13.7)
Preoperative weight loss in kg, mean (SD)	5.7	(4.4)
Medical history		
Dyslipidemia, *n* (%)	120	(51.9)
NASH, *n* (%)	109	(47.2)
Hypertension, *n* (%)	124	(53.6)
Obstructive sleep apnea, *n* (%)		
Mild (AHI, 5–14.9 events per hour)	59	(25.5)
Moderate (AHI, 15–29.9 events per hour)	43	(18.6)
Severe (AHI, ≥30 events per hour)	77	(33.3)
Type 2 diabetes, *n* (%)	67	(29)
Coronary heart disease, *n* (%)	3	(1.3)
Laboratory data before surgery		
HbA1c level, % (SD)	6.11	(1.18)
Glucose level, mg/dL (SD)	102.7	(19.4)
Total cholesterol level, mg/dL (SD)	189.6	(38.3)
HDL cholesterol level, mg/dL (SD)	168.5	(85)
Triglycerides level, mg/dL (SD)	45.9	(12.1)
25‐Hydroxy Vitamin D, ng/dL (SD)	25.8	(8.9)
Type of surgery		
RYGB, *n* (%)	140	(64.7)
Sleeve, *n* (%)	75	(35.2)
Mini‐gastric bypass, *n* (%)	16	(6.9)
Adherence to diet	128	(55.4)
Continued follow‐up with a nutritionist	165	(71.4)
Number of visits with the nutritionist, median (IQR)	7	(5–9)
Physical activity after surgery		
>30 min/day, *n* (%)	161	(69.7)
>5 days/week, *n* (%)	93	(40.2)
Mild intensity, *n* (%)	47	(20.3)
Moderate and vigorous intensity, *n* (%)	177	(76.6)
Alcohol consumption ≥25 g after surgery, *n* (%)	39	(16.8)
Anthropometric measures after surgery		
%TWL after 1 year of follow‐up, mean (SD)	32	(6.5)
%EWL after 1 year of follow‐up, mean (SD	83.2	(18.9)
BMI at the end of follow‐up in kg/m^2^, mean (SD)	27.9	(3.3)

Abbreviations: AHI, apnea‐hypopnea index; BMI, body mass index; HbA1c, glycated hemoglobin A1c; HDL, high‐density lipoprotein; IQR, interquartile range; NASH, non‐alcoholic steatohepatitis; RYGB, laparoscopic Roux‐en‐Y gastric bypass; SD, standard deviation; TWL, percentage of total weight loss; %EWL, percentage of excess weight loss.

The mean baseline BMI was 41.4 ± 5.1 kg/m^2^, and 1 year after bariatric surgery, it was reduced to 27.9 ± 3.3 kg/m^2^ (mean difference 13.2, *p* < 0.001). A similar finding was evident when the basal BMI was compared with the BMI at 2 years (mean difference 13.2, *p* < 0.001) and 3 years after surgery (mean difference, 14.1; *p* < 0.001). The waist perimeter decreased from 124.0 ± 13.2 cm at baseline to 98.1 ± 10.9 cm at 1 year (*p* < 0.001).

**FIGURE 1:**
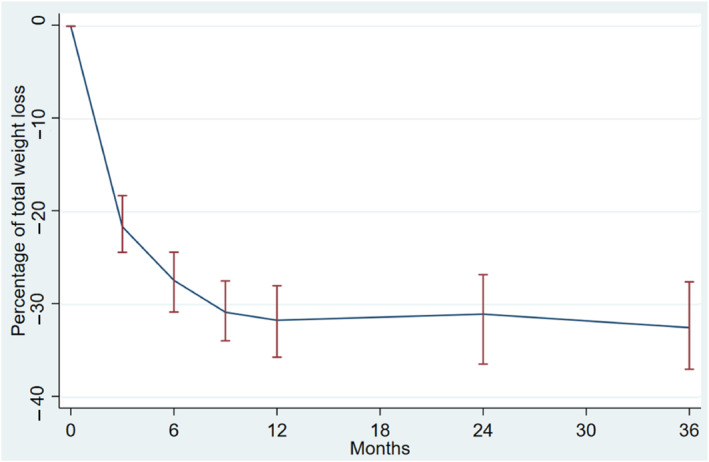
The weight change as a percentage of total weight loss during the 36‐month follow‐up period.

Follow‐up was performed at 3 (*n* = 141), 6 (*n* = 208), 9 (*n* = 115), 12 (*n* = 216), 24 (*n* = 166), and 36 (*n* = 99) months. There was a tendency for patients to lose weight in the first 12 months and then stabilize thereafter. The %TWL increased after 6 (27.5 ± 5.6%), 9 (30.9 ± 5.9%), and 12 months (31.7 ± 6.1%), and then tended to stabilize after 24 (31.4 ± 6.1%) and 36 months (32.8 ± 6.1%) (Figure 1). Only 6 (3.2%) patients had a %TWL <20% after 12 months of follow‐up.

The GEE model allowed us to analyze different patterns of weight change over time by using the %TWL. The longitudinal analysis showed an average increase of 0.7% per month of follow‐up (*p* < 0.001). On average, patients with a basal BMI ≥40 kg/m^2^ had a higher increase in the %TWL than those with a lower BMI (0.4%, *p* < 0.001). Similarly, patients who lost ≥10 kg before surgery had a higher increase in the %TWL than those who lost <10 kg (0.3%, *p* = 0.001). Patients with continued follow‐up with a nutritionist increased their %TWL on average by 2.3% (*p* = 0.004) compared with patients without follow‐up (Table 2).

**TABLE 2:** Generalized estimating equation analysis showing the factors affecting changes in %TWL after bariatric surgery.

Factor	%TWL		
	Coefficient	95% CI	*p*‐value
Time (months)	0.7	0.6, 0.7	<0.001
Baseline BMI >40 kg/m^2^ ^a^	0.4	0.3, 0.5	<0.001
WL before surgery >10 kg^b^	0.3	0.1, 0.4	0.001
Physical activity >30 min/day^c^	−0.7	−1.1, 0.2	0.002
Continued follow‐up with a nutritionist^d^	2.3	0.7, 3.9	0.004

Abbreviations: BMI, body mass index; CI, confidence interval; WL, weight loss; %TWL, percentage of total weight loss.

^a^ Comparison with patients with a BMI <40 kg/m^2^.

^b^ Compared with patients with WL before surgery <10 kg.

^c^ Compared with patients with physical activity <30 min/day.

^d^ Compared with patients who continued follow‐up with a nutritionist <2 times per year.

Patients who performed >30 min of physical activity had a lower %TWL by 0.6% (*p* = 0.002) (Table 2). Those ≥65 years of age had less WL than younger patients, without achieving a statistically significant difference (*p* = 0.06). No association was found between adherence to diet, anxiety, binge eating disorder before surgery, the type of surgery, alcohol consumption, the total number of visits, and other clinical variables, such as a history of TD2 and the HbA1c level.

## 4 DISCUSSION

Baseline BMI >40 kg/m^2^, WL before surgery >10 kg, continued follow‐up with a nutritionist, and time of physical activity during the 12 months after surgery were associated with a significant change in the %TWL. Multiple guidelines have described several predictors of successful postoperative WL, such as behavioral changes, preoperative WL, and nutrition therapy.^19^ Similar to other cohort studies, this study showed a tendency for the %TWL to increase in the first year and then plateau thereafter.^20^, ^21^

Although the age and proportion of women in this study were similar to those reported in other cohort studies, baseline BMI was significantly lower.^22^ In Latin American countries such as Brazil^23^ and Mexico,^4^ the basal BMI values were 49.3 ± 8.3 kg/m^2^ and 48 kg/m^2^, respectively. This finding highlights the importance of analyzing and reporting data from different regions. Preoperative BMI has an important effect on WL outcomes among patients who underwent bariatric surgery.^20^, ^21^ Seo et al. reported that the increase in the %TWL was higher in patients treated with RYGB whose preoperative BMI was ≥40 kg/m^2^ than in their counterparts, and it kept increasing up to 5 years after surgery.^20^

The patients included in this study were evaluated by a nutritionist, psychiatrist, and endocrinologist prior to surgery. These assessments were aimed at diagnosing and treating pathologies related to obesity, modifying dietary habits, and identifying eating disorders prior to surgical intervention. The pre‐surgical WL reported in this study is similar to that reported in other studies that implemented a combination of interventions such as diet, physical activity, and behavior changes.^24^ Although information about the simultaneous use of these interventions is conflicting, this approach has no significant impact on postoperative WL, as reported previously.^24^ However, it has a positive effect on postoperative depression and dysfunctional eating patterns as well as obesity‐related comorbidities, for example, diabetes, hypertension, and dyslipidemia.^24^

The benefit of preoperative WL on long‐term postoperative WL remains unclear.^25^ However, patients are required to lose weight before surgery to reduce surgical complications in most of the obesity clinics. Recently, Sun et al. analyzed the association of WL and mortality 30 days after bariatric surgery in 480,075 patients^26^ and found that a small preoperative WL was associated with a statistically significant reduction in postoperative mortality,^26^ suggesting that the preoperative WL should be “goal‐oriented” without delaying the surgical intervention.^25^ In this study, WL ≥ 10 kg was associated with a better %TWL at the end of follow‐up than WL < 10 kg.

This study reported continued follow‐up with a specialized nutritionist as the main predictor of the %TWL. Interestingly, continuous visitation over time was more important than the number of visits per se. In a similar way, Tewksbury et al. found that the frequency of virtual or face‐to‐face contacts during follow‐up after bariatric surgery was not associated with postoperative WL.^27^ This finding suggests that it is more important to maintain sustained tracking over time than a large number of visits at the start without persistent tracking. Although 81% of patients were adherent to nutritional follow‐up, only 55.6% of the included patients were adherent to the dietary recommendations. Other studies have reported that adherence to the postoperative diet, after participants have returned to eating regular foods, was also associated with larger postoperative WL.^28^, ^29^ Regular nutritional follow‐up and compliance with postoperative dietary counseling have been reported to improve weight maintenance.^30^ Additionally, compared with patients without continued follow‐up, those with continued follow‐up had better adherence to multivitamin supplementation, reducing the risk of nutritional deficiencies following bariatric procedures^30^; such compliance will minimize nutritional deficiencies that cause fatigue, minimize food cravings to improve long‐term WL, and provide increased quality of life.^31^ The consumption of 30 g/day of alcohol, regardless of the type of liquor, is associated with weight gain and obesity.^23^ However, alcohol consumption was <30 g in this population, and no association was found.

Exercise improves health, with well‐characterized physiological and WL benefits, including WL maintenance.^32^ A previous study reported that in patients treated with bariatric surgery and followed for 5 years, increased time of physical activity helped to maintain the WL.^33^ Several studies have suggested that moderate to vigorous intensity physical activity of ≥150 min/week is critical for maintaining WL.^32^, ^34^, ^35^ Nevertheless, the patients in our study who exercised ≥30 min had less %TWL. The physical activity data were self‐reported by the patients in this study, and previous publications have reported that the patients' perception of physical activity was greater after bariatric surgery; however, when the objective measurement was made, up to 29% of the patients were less active in the postoperative period than in the preoperative period. The information from this study does not allow us to conclude that changes should be made in the physical activity recommendations in this population; therefore, additional studies are required in this respect.

As a strength, these data showed factors related to a significant change in the %TWL and allowed us to quantify its impact over time in patients treated with bariatric surgery who received usual care during a long‐term follow‐up at an obesity clinic with a multidisciplinary team. Similarly, biochemical and clinical factors such as diet, exercise (time, intensity, and frequency), and alcohol consumption were recorded and analyzed. Among limitations, patient loss to follow‐up was significant after 12 months of follow‐up, which may have been related to the termination of coverage by the insurance company. This factor may have affected the accuracy of the estimates at that time. However, the losses in the first 12 months, when significant changes in weight were observed, were minimal. This finding reflects the real‐life care of patients treated with bariatric surgery. Moreover, body composition data were not available for this study. Lastly, eating behaviors such as binge eating or uncontrolled eating were not evaluated during the follow‐up; however, all patients had a preoperative nutritional and psychiatric evaluation that ruled out this type of behavior before surgery.

## 5 CONCLUSIONS

Patients have a tendency to increase the %TWL during the first 12 months after bariatric surgery, with weight stabilizing after that time. Establishing the factors associated with early weight change reinforces the importance of postoperative follow‐up in this population. Modifiable factors such as continued follow‐up with a nutritionist and WL of ≥10 kg before surgery improves the weight change are different from the baseline BMI and physical activity >30 min/day, as these factors are associated with a significant decrease in the %TWL. These factors should be evaluated during the early stage after bariatric surgery. Additional studies are required to clarify other factors associated with WL, such as patterns of physical activity during the long‐term follow‐up.

## AUTHOR CONTRIBUTIONS

Diana C. Henao, Ana M. Gómez and Oscar M Muñoz conceptualized the research question and wrote the initial version of the manuscript. Claudia Rubio, Natalia Rodríguez and Valentina Ursida completed the data collection. Oscar M Muñoz performed the data analyzes. All authors designed the study, interpreted the data, critically reviewed the manuscript, and approved the final version as submitted.

## CONFLICT OF INTEREST STATEMENT

Diana C. Henao reports speaker fees from Novo Nordisk, Abbott, and Medtronic. Ana M. Gomez reports speaker fees from Novo Nordisk, Eli Lilly, Boehringer Ingelheim, Abbott, and Medtronic. Oscar Muñoz reports research grants from Novo Nordisk. The other authors declare no potential conflicts of interest.
